# Altered rainfall patterns increase forb abundance and richness in native tallgrass prairie

**DOI:** 10.1038/srep20120

**Published:** 2016-02-01

**Authors:** Sydney K. Jones, Scott L. Collins, John M. Blair, Melinda D. Smith, Alan K. Knapp

**Affiliations:** 1 Department of Biology, MSC03-2020, University of New Mexico, Albuquerque, NM, 87131, United States; 2 Division of Biology, Kansas State University, Manhattan, KS, 66506, United States; 3 Graduate Degree Program in Ecology, Colorado State University, Fort Collins, CO, 80523, United States; 4 Department of Biology, Colorado State University, Fort Collins, CO, 80523, United States

## Abstract

Models predict that precipitation variability will increase with climate change. We used a 15-year precipitation manipulation experiment to determine if altering the timing and amount of growing season rainfall will impact plant community structure in annually burned, native tallgrass prairie. The altered precipitation treatment maintained the same total growing season precipitation as the ambient precipitation treatment, but received a rainfall regime of fewer, larger rain events, and longer intervals between events each growing season. Although this change in precipitation regime significantly lowered mean soil water content, overall this plant community was remarkably resistant to altered precipitation with species composition relatively stable over time. However, we found significantly higher forb cover and richness and slightly lower grass cover on average with altered precipitation, but the forb responses were manifest only after a ten-year lag period. Thus, although community structure in this grassland is relatively resistant to this type of altered precipitation regime, forb abundance in native tallgrass prairie may increase in a future characterized by increased growing season precipitation variability.

Changes in plant community structure and function are generally driven by multiple biotic and abiotic factors, such as resource competition, nutrient availability, precipitation patterns and disturbance regimes. The rate, pattern, and intensity of many of these drivers are rapidly changing under global environmental change, including increased climate variability, elevated levels of atmospheric N deposition, and changes in the type, frequency and intensity of disturbances^1^,^2^,^3^,^4^. As a consequence of altered biotic and abiotic drivers, plant communities are likely to undergo significant changes in ecological structure and function under global environmental change.

Plant community structure and ecosystem functioning of mesic grasslands, for example, are strongly driven by several factors, including grazing and fire^3^,^5^,^6^, nutrient availability^7^,^8^,^9^, and precipitation amount and variability^10^,^11^,^12^,^13^,^14^,^15^. Indeed, aboveground net primary production (ANPP) of mesic tallgrass prairie has been shown to be limited by both water and nitrogen availability^7^,^16^,^17^. Similarly, persistent drought reduced ANPP in mesic grasslands, but effects on plant species richness and community composition were variable and inconsistent^18^,^19^,^20^. Thus, changes in water availability and variability may have relatively consistent impacts on grassland ANPP but more variable effects on community composition and structure.

In the US Great Plains, predicted changes in total annual precipitation are inconsistent under different future climate change scenarios although soils are predicted to become drier due to elevated temperatures^21^. In addition, models consistently predict an intensification of the hydrological cycle with increased interannual variability in rainfall and, possibly of greater importance, altered within-season rainfall patterns such that rain events will be fewer but larger with longer intervals between rains^22^,^23^,^24^. Indeed, the number of extreme precipitation events in the US Great Plains has increased markedly during the past decade^25^,^26^. The ecological impacts of variation in total seasonal precipitation amount verses within-season patterns of rainfall remain a subject of considerable uncertainty and debate^27^,^28^.

Evidence suggests that changes in precipitation regimes during the growing season may have significant ecological consequences for grassland structure and function, and that these effects are mediated primarily by altered soil moisture availability and by increased duration and frequency of dry periods between rain events^28^,^29^,^30^,^31^. Indeed, increased rainfall variability in mesic grassland, independent of amount, has been shown to reduce ANPP, soil respiration, and leaf level photosynthesis over the short term, and to alter the genotypic diversity of the dominant grasses over longer time frames^1^,^4^,^32^,^33^,^34^,^35^,^36^. Together these results support predictions that grassland ecosystems will be highly responsive to future changes in precipitation variability.

Mechanisms controlling ecosystem responses to altered resource availability vary among grasslands. For example, Hallett *et al.*^37^ found strong species asynchrony, where decreases in abundance of one species are compensated for by increases in others, in sites characterized by high precipitation variability, whereas species richness and dominance reduced temporal variability in sites with high mean annual precipitation. This is consistent with Fay *et al.*^31^ who reported that dominant prairie grasses were generally buffered against more variable precipitation regimes. However, Hautier *et al.*^38^ found that increased soil nutrient availability (plant available nitrogen) decreased the effectiveness of these stabilizing mechanisms in grasslands. It is unclear, therefore, if changes in soil moisture availability will have similar effects on grassland community stability as altered soil nutrient availability.

The Hierarchical Response Framework (HRF) predicts that chronic changes in resource availability, such as soil moisture, will eventually lead to reordering of species abundances within communities, and ultimately species replacement over time, often but not always after some time lag has occurred^39^. For example, mesic grassland communities responded rapidly to nitrogen addition, but slowly to increased soil moisture availability^40^,^41^,^42^. Because rate and pattern of response to long-term (chronic) alterations in resources varies considerably among ecosystems short-term outcomes may not accurately reflect long-term responses to chronic changes in resource availability. Yet, understanding how grassland communities respond to long-term changes in resource variability is important because changes in species composition can, in turn, feedback to alter ecosystem functions, such as ANPP^14^,^40^,^43^. Therefore, we investigated how long-term changes in *intra*-annual precipitation variability, which drive patterns of soil moisture availability, affected long-term dynamics of plant community composition and structure in native tallgrass prairie.

We used data from a unique 15-year long rainfall manipulation experiment at the Konza Prairie Biological Station in northeastern Kansas, USA, to determine how altered precipitation patterns (fewer, larger events) impacted plant species composition and structure in an annually burned, ungrazed, native tallgrass prairie. We tested two hypotheses. First, based on the HRF, we predicted that directional change in grass and forb cover and richness and community composition would eventually occur after a lag period under the altered precipitation treatment. Second, we predicted that change in cover and composition under altered precipitation would be driven by the response of forbs more so than grasses because the dominant grasses are reported to be buffered against precipitation variability^1^,^44^ and changes in the cover and richness of forbs contribute disproportionately to community responses to other drivers in this grassland^3^,^5^,^6^.

## Results

### Rainfall Regime

**Figure 1:**
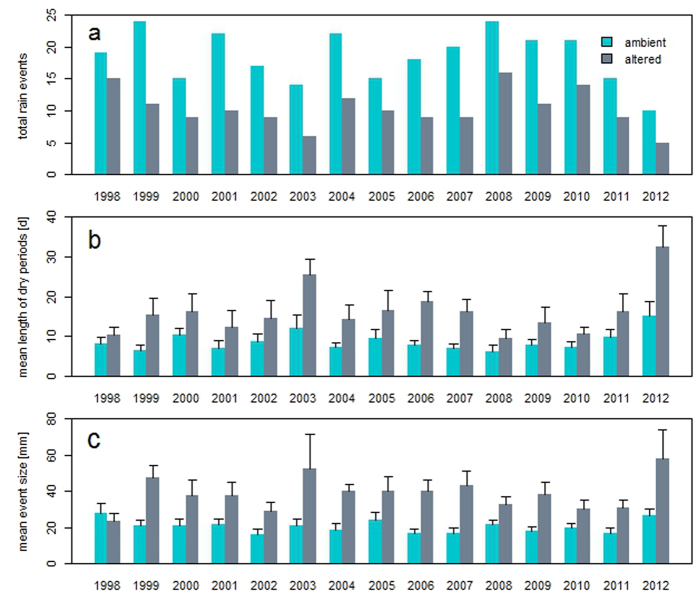
Growing season precipitation parameters between ambient and altered treatments (fewer, larger rain events) from 1997 (pretreatment year) through 2012. (**a**) total number of rain events excluding events less than 5 mm. (**b**) mean number of days between rain events or the mean length of dry periods in days. (**c**) mean size of rain events (mm). Differences between ambient and altered treatments were significant (*P* = < 0.0001) for all parameters. Error bars denote one standard error.

Growing-season rainfall averaged 404.0 mm (±2.0 se) from 1997 through 2012. From May through September an average of 29 ambient rainfall events occurred per year, with an average of 18 events >5 mm. The altered precipitation treatment resulted in an average of 12 rainfall events per year, with 10 events >5 mm (Fig. 1a). Mean length of dry periods between events averaged 8.7 (±1.9 se) days for ambient and 16.2 (±3.7 se) days for altered treatments (Fig. 1b). The nearly 100% increase in mean length of dry periods in altered compared to ambient treatments was due to the removal of multiple small rain events, which were most frequent on average during the months of May, June and August. Mean event size averaged 20.6 mm (±3.3 se) and 38.8 mm (±7.4 se) for ambient and altered precipitation treatments, respectively (Fig. 1c).

### Soil Moisture Patterns

Seasonal mean soil water content (SWC) was significantly (5.3%) lower in the altered (23.0% ± 2.3 se) compared to ambient (24.3% ± 2.3 se) treatment during the growing season (*P* < 0.0001; Fig. S1a). This difference was greatest during the first six years of the experiment. In contrast, mean soil moisture was similar in the altered compared to the ambient treatment in the last three years of the experiment when below average precipitation occurred. The difference between ambient and altered SWC was marginally related to total ambient precipitation (*R*^*2*^ = 0.24, *P* = 0.0787; Fig. S1b). The 15-year growing season mean SWC in the altered treatment was below that of ambient mainly during August and September. The 15-year mean growing season deviation from ambient was 1.5% (±2.6 se), the mean maximum SWC was 3% lower and the duration of low SWC (20% or lower) was 19 days (38.7%) longer in the altered treatment compared to ambient. The 2005 growing season, a representative year showing a similar pattern, had a SWC mean deviation from ambient of 1.7%, a maximum value of about 6% lower and the duration of low SWC was longer by 14 days (78.8%) in the altered compared to ambient treatment (Fig. S2). Coefficient of variation (CV) of SWC was higher, on average, in the altered (44.3 ± 2.4 se) compared to ambient (37.4 ± 2.0 se) treatment over this 15-year period.

### Plant Responses

**Figure 2:**
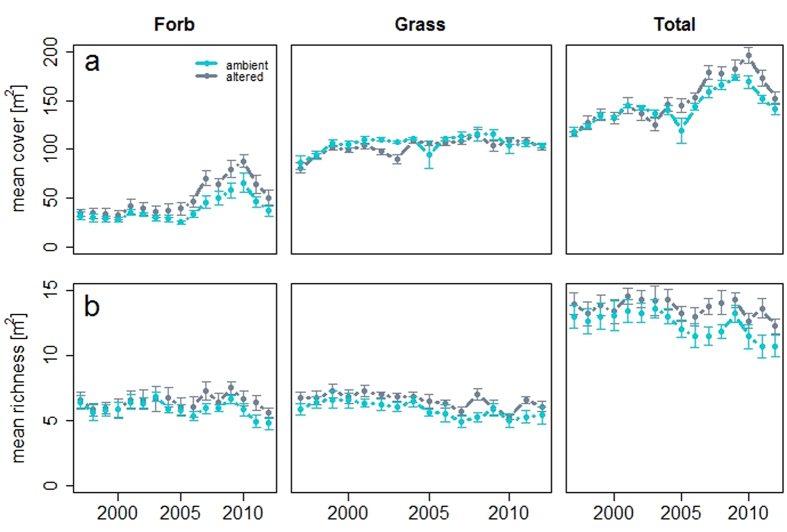
(**a**) Forb, grass, and total cover by treatment from 1997–2012. Total cover was higher on average under the altered (fewer, larger rain events) treatment, primarily due to an increase in forb cover rather than grass cover. However, this effect did not emerge until after ten years of altered precipitation. (**b**) Forb, grass, and total richness by treatment from 1997–2012. Total and grass richness were consistently higher on average in the altered treatment over the duration of the experiment. Higher altered forb richness occurred after a lag of ten years. Error bars denote one standard error.

Relative to the ambient treatment, 15 years of increased rainfall variability resulted in generally higher average total cover and species richness (Fig. 2), but these differences were driven more by changes in forbs than grasses. Total cover was higher on average by 5.5% under altered precipitation, primarily due to an overall 24% increase in forb cover, and slight decline in mean grass cover. Forb cover (30.5%, P < 0.001) and richness (8.9%, P = 0.008) were significantly higher under altered precipitation compared to the ambient. These changes were first observed around 2007, ten years after the start of the experiment (Fig. 2). Grass cover in the altered treatment was lower than ambient, but only by 3.4% (P = 0.07, Table 1) due to strong declines in 2002 and 2003 when precipitation was below average. Grass and total richness were also higher in the altered compared to the ambient treatment by 12.2% and 10.5% respectively (Table 1), but the difference in grass richness existed prior to the start of the experiment. Forb richness was positively correlated to average seasonal SWC in ambient treatments (r^2^ = 0.42, P = 0.013) and nearly so under altered precipitation (r^2^ = 0.20, P = 0.105). Total, grass and forb richness were unrelated to total seasonal rainfall, average days between rainfall events, and CV of SWC in both ambient and altered treatments (Table S1).

**Figure 3:**
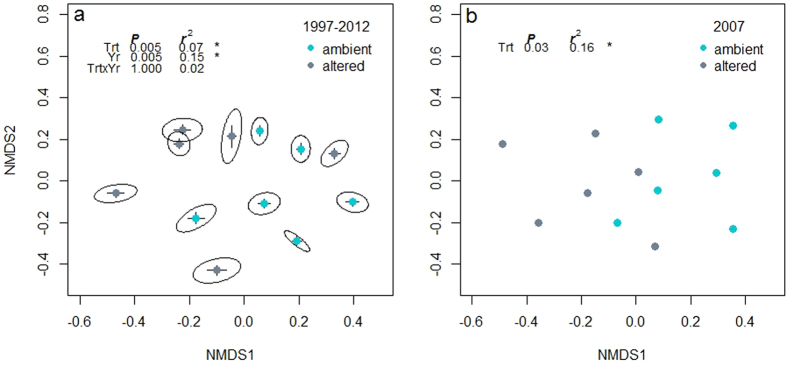
Non-metric multidimensional scaling (NMDS) of plant community composition for all plots (**a**), between 1997 and 2012 and (**b**), 2007 only, the year altered forb cover increased significantly from ambient. Each point is the temporal average of the NMDS coordinates for a given RaMP (plot) over the 15-year period. Error bars are one standard error. Ellipses are 95% confidence clouds based on standard error. Treatment is indicated by color. Ambient treatments are plots in which precipitation was added immediately after a natural rain event and altered treatments are plots with fewer, larger rain events. Annual treatment differences were only significant in 2007 and 2009 based on the PERMANOVA.

Based on time-lag analysis, directional change occurred at similar rates under ambient (mean slope = 2.84) and altered (mean slope = 2.96) treatments (Table 2). This is reflected in the lack of distinct spatial separation between treatments in the NMDS spanning the full experimental time (Fig. 3a). Based on PERMANOVA, however, significant differences in community composition between ambient and altered treatments occurred in only two of 15 years (2007 and 2009) which resulted in an overall treatment effect on community composition across all years (Fig. 3a, inset). Indeed, this is seen in the NMDS for year 2007 where there is significant separation of ambient and altered treatments (Fig. 3b, inset). Population stability (averaged temporal variability of each species over time) and community stability (aggregated species abundance) were not significantly different between altered and ambient rainfall regimes (Table 2). Mean rank shift, relative changes in species rank abundances, also did not differ significantly among treatments (Table 2).

Three grasses (*Andropogon gerardii*, *Sorghastrum nutans*, and *Andropogon scoparius*) and two common forb species (*Solidago canadensis* and *S. missouriensis*) accounted for 64% of the difference in community composition between ambient and altered rainfall treatments over the 15 years based on the SIMPER analysis (Table 3). In particular, the dominant grasses *A. gerardii* and *S. nutans* had lower abundance, while *A. scoparius*, a perennial caespitose species more characteristic of drier sites, and *S. missouriensis*, a clonal understory forb, had higher abundance in altered relative to the ambient treatment. A similar pattern occurred in the 2007 SIMPER analysis. In 2009, *S. nutans* explained most of the community differences between rainfall treatments with *A. scoparius* second in importance, along with the perennial forb *Helianthus rigida* (Table 3).

## Discussion

We found that increasing the size of rainfall events while reducing the number of events resulted in relatively limited changes in plant community composition and structure in this annually burned, ungrazed, tallgrass prairie. As we predicted, the altered precipitation regime eventually resulted in higher levels of forb cover and richness, while grass cover remained relatively stable over time. Richness of grasses and forbs was higher under altered precipitation by 1-2 species m^−2^ on average. Directional change in community composition occurred under both ambient and altered precipitation regimes, possibly in response to annual burning^45^, as was observed in an adjacent annually burned, long-term irrigation experiment^41^. Species richness is known to be negatively correlated with fire frequency in this system^46^. Thus, annual burning is likely driving the overall decline in species richness and explains why we found a strong year effect on both grass and total richness. Nevertheless, the greater increase in forb cover and richness under the altered precipitation regime in this experiment ultimately led to significantly different species assemblages; however, these differences resulted from changes in abundance of species already present in the altered treatment plots, rather than in new species establishing in the community.

Altering the amount and duration of rain events resulted in significantly lower and more variable soil water content than under ambient precipitation, and this was predicted to influence community composition and dynamics. Results from modeling studies and rainfall manipulation experiments in tallgrass prairie show that changes in the temporal patterns of soil water content have consequences for numerous ecosystem processes that can affect plant community composition^27^,^32^,^34^,^44^,^47^. Precipitation variability, expressed through soil moisture availability, is a well-documented driver of grassland community dynamics from one year to the next, but community sensitivity to precipitation variability may be contingent upon preexisting environmental contexts^48^,^49^ and variable mechanisms of community stability^37^. For example, Cherwin and Knapp^34^ found high variation in productivity responses of shortgrass steppe sites to experimentally imposed drought, despite consistent reductions in soil moisture. In a modeling study, Gerten *et al.*^27^ found productivity responses to precipitation change to be determined by current degree of water limitation. Overall, these generally short-term manipulations led to rapid initial responses that may not adequately represent community dynamics that may play out over a decade or more.

The “bucket” model predicts that increased precipitation variability resulting from fewer rain events and longer duration of dry periods will increase the duration of soil water stress in mesic grasslands^28^. However, larger rain events will allow deeper infiltration of soil moisture to the benefit of deeper-rooted forbs compared to fibrous rooted grasses. Thus, although the bucket model predicts that productivity will be negatively affected by increased precipitation variability, such alterations in precipitation regimes may have different impacts on the plant community. In a short-term study, Heisler-White *et al.*^33^ found that fewer, larger rain events in a mesic grassland resulted in an 18% decrease in ANPP but no significant change in forb cover or richness. We also found lower grass cover under altered precipitation patterns in our study, but the increase in forb abundance and richness took nearly 10 years to develop illustrating how short-term responses may not reflect long-term dynamics, and highlighting the need for more long-term climate manipulation experiments^50^.

The eventual rise in forb cover under the altered precipitation regime is likely linked to changes in soil water content at depth^28^,^51^ and can potentially be explained by the differential use of soil water between grasses and forbs, although evidence in support of this hypothesis is inconsistent. Nippert and Knapp^51^ found that C_4_ grasses in tallgrass prairie consistently relied on shallow soil water (5 cm) across the growing season while C_3_ forbs also utilized deeper soil layers when shallow layers were depleted, supporting the hypothesis of soil water partitioning between grasses and forbs when shallow soil moisture is depleted. Because fewer, larger rainfall events, such as those imposed here, promote deeper water infiltration and extended drying of surface soils, resource partitioning between shallow and deep soils may contribute to increased forb abundance in this system. Indeed, Hoover *et al.*^20^ found that differences in rooting depth between *S. canadensis* and C_4_ grasses caused *S. canadensis* to use deeper soil moisture during a 2-year drought experiment. Conversely, Fay *et al.*^44^ found that photosynthetic responses of *S. canadensis*, a species that accounted for 13% of variation in compositional differences between treatments in our study, were positively correlated with increased shallow soil moisture variability, suggesting that this species may at times also rely more on shallow than deep soil moisture. Furthermore, Koerner *et al.*^52^ found a strong negative correlation between grasses and forbs in this system. When grass cover increased due to herbivore removal, forb richness decreased. The tall dominant grass *A. gerardii*, whose cover decreased substantially under the altered precipitation regime, most likely competes with forbs for light and nutrient resources as well as water. The reduction in cover of *A. gerardii* in combination with changes in temporal soil water content likely combined to increase forb cover.

The lagged response in forb cover is consistent with the temporal hierarchy of ecological responses to chronic changes in resource availability predicted by the Hierarchical Response Framework^39^. For example, the persistence of dominant species could have a disproportionate influence on overall species turnover and community dynamics in grasslands^37^,^53^. The dominant C_4_ grasses in our study exhibited little change in abundance over the 15 years of precipitation treatments. However, these consistent measures of abundance could mask subtle changes in genotype diversity occurring within the dominant species. Specifically, long-term alteration of precipitation variability in this experiment has led to a shift in the genotypic composition of the dominant perennial grass, *A. gerardii*^35^, and the changes in these genotypes reflect adaptations to a more variable precipitation regime^54^. Thus, overall stability in the dominant grasses regardless of the increase in forb abundance may result from genotypic divergence within the long-lived dominant C_4_ grasses induced by chronic changes in soil moisture availability.

The overall low levels of community and population variability and lagged response of forbs found in this study suggest that the rate of species turnover in this tallgrass prairie may be mitigated, in part, by genetic diversity and functional traits of dominant grasses, at least until some threshold in resource availability is reached. The overall high level of community stability and low rate of temporal change in community composition may result from an increase in cover and richness of a few forb species along with a concomitant decrease in other species, resulting in no net change in abundance, and thus high community stability. Thus, stability is driven by compensatory dynamics (negative covariance) in which trade-offs among species populations stabilize the overall community^37^ as a consequence of differential responses to environmental variation. Moreover, the magnitude of precipitation variability we imposed may have crossed the resource limitation threshold of only a few species leading to overall community stability and lower demographic stochasticity^55^.

Although chronic changes in resource availability are predicted to significantly alter ecosystem structure and function^39^, this long-term study suggests that mesic tallgrass prairie is relatively resistant to long-term increases in intra-annual precipitation variability. Our findings are in line with other recent studies showing relatively high resistance to climate manipulations^56^,^57^, such as heat waves and increased precipitation. For example, Hoover *et al.*^20^ imposed heat waves combined with extreme drought on mesic grassland during two consecutive growing seasons. Although ecosystem function was significantly impaired during the drought, ANPP was resistant to this combination of extremes. Collins *et al.*^41^ found that nineteen years of irrigation led to few changes in grassland community structure and function despite a significant increase in net primary production^14^. In this study, our manipulation of rainfall variability, did not reduce total precipitation during the growing season, but resulted in an unprecedented change in the rainfall regime^58^,^59^. Thus, this mesic tallgrass prairie is an example of an ecosystem that is both resistant to long-term changes in precipitation variability and amount, as well as relatively resilient to short-term extremes, with community stability strongly influenced by high resistance of the dominant grass species to these forecast alterations in climate.

## Methods

### Study Site

This study was conducted in the Rainfall Manipulation Plots (RaMPs) experiment at the Konza Prairie Biological Station (KPBS), a 3487 ha native tallgrass prairie preserve and Long-Term Ecological Research (LTER) site located in the Flint Hills region of northeastern Kansas, USA. Konza Prairie is characterized by a temperate mid-western continental climate with a mid-growing season mean temperature of 27 °C and mean annual precipitation of 835 mm y^−1^, of which 75% occurs during the growing season (May through September^32^). Growing season rainfall generally declines from June through September and is somewhat bimodal with high rainfall periods between May and June, and another smaller period in September. July through August has lower rainfall with high temperatures^41^,^60^. Yearly and seasonal variation from these general precipitation patterns is common^61^.

The RaMPs are located in typical lowland prairie on Irwin silty clay loam soils around 320 m above sea level. Vegetation is a matrix of perennial, warm season (C_4_) tall grasses and primarily perennial C_3_ forbs. The dominant grasses include *Andropogon gerardii A. scoparius*, *Panicum virgatum*, *Sorghastrum nutans*, and *Sporobolus asper*. Common forb species include *Aster* spp., *Ambrosia psilostachya*, *Helianthus rigida*, *Solidago* spp., *Kuhnia eupatoroides*, *Salvia azurea* and *Vernonia baldwinii*^41^. The plots are burned annually each spring in late March, a burn regime characteristic of management practices throughout the region.

The RaMPs facility consists of 12 fixed rainout shelters located on 14 × 9 m experimental plots and three unsheltered control plots. Each plot includes a 6 × 6 m sampling area enclosed within a perimeter of sheet metal that extends 0.1 m above ground and 1.1 m deep. During the growing season, shelters are covered with polyethylene roofing, diverting natural rainfall into gutters that lead into storage tanks. Rainfall is then reapplied with an overhead irrigation system. Plots are instrumented with Campbell CSR-616 TDR probes to measure soil volume water content at one minute intervals averaged for each day. More details on the experimental design and instrumentation are provided in Fay *et al.*^60^.

### Rainfall Manipulation Treatments

RaMPs shelters allowed for (1) an ambient treatment (*n* = 6) that applied rain immediately (within 24 h) after natural rainfall events, replicating the natural precipitation regime and (2) an altered treatment (*n* = 6) that applied identical amounts of rainfall as ambient plots totaled across the growing season, but lengthened the time between rainfall events by 50%, resulting in fewer but larger events with longer dry intervals between events^31^. Treatments were applied during every growing season from 1998–2012, with 1997 serving as a pretreatment year. Experimental rainfall applications each year were based on ambient rainfall measurements via on-site rain gauges. Unsheltered control plots (*n* = 3) were used to assess shelter effects, which have been reported previously^60^ and are not analyzed here. Frequency and amount of rain events and soil moisture were measured throughout the duration of the experiment.

### Measurements of Plant Community Structure

Each RaMP had four contiguous 1 m^2^ permanent quadrats in a 2 × 2 m grid in which plant species composition was measured spring and fall every year by visually estimating percent cover for all plant species in each quadrat. Species richness (cumulative number of species m^−2^ appearing in spring and fall samples) was determined each year in each quadrat. Richness and cover in each quadrat were averaged across the four quadrats to get mean richness and cover per RaMP. Repeated measures analysis of variance (RM-ANOVA) was used on total, grass and forb cover and richness data to assess main and interactive effects of treatment and year, with year as a repeated factor. Cover data were arcsine square root transformed to improve normality. Cover data were used further to evaluate community composition dynamics. Non-metric multidimensional scaling (NMDS) was used to visually determine trajectories of change of community composition over time among replicates of each rainfall treatment. Permutational multivariate analysis of variance (PERMANOVA) using Bray-Curtis dissimilarities was performed on community data to assess differences in community composition resulting from main effects (treatment and year) and their interactions. All analyses were performed on the mean values based on the average of the four 1m^2^ quadrats in each RaMP.

To assess potential directional change in community composition under altered precipitation, we used time-lag analysis to quantify the rate of change in community composition in each RaMP over the 15 year study period. Time-lag analysis uses Euclidean distance to measure similarity of community samples at increasing time lags and when regressed, yields an estimate of rate of change over time^62^,^63^. To assess potential differences in temporal variability between treatments at the population and community level, population and community stability were calculated. *Population stability* is the average of the temporal variability (mean to variance ratio) of each species over time^64^, and *community stability* is the temporal variability (mean to variance ratio) of total cover (aggregated species abundances) over time. Community stability was evaluated for the whole community, as well as for forb and grass functional groups separately. Larger values indicate greater temporal stability. Species mean rank shift was also calculated per treatment. This measure quantifies relative changes in species rank abundances by taking the sum difference of species ranks of consecutive pairs of years^65^. We used analysis of variance (ANOVA) and Tukey’s HSD multiple comparisons test to assess treatment effects on population and community stability and mean rank shifts. Finally, to determine changes in the relative contribution of forb species to community composition under altered precipitation, similarity percentages (SIMPER) using Bray-Curtis dissimilarities were calculated between treatments. Again, all analyses were performed on the average of the four 1m^2^ quadrats in each RaMP. Statistical calculations were conducted using R^66^ and the R package “vegan”^67^.

## Additional Information

**How to cite this article**: Jones, S. K. *et al.* Altered rainfall patterns increase forb abundance and richness in native tallgrass prairie. *Sci. Rep.* **6**, 20120; doi: 10.1038/srep20120 (2016).

## Supplementary Material

Supplementary Information

## Figures and Tables

**Table 1:** Repeated measures analysis of variance for total, grass, and forb cover and richness in ambient and altered precipitation treatments in mesic tallgrass prairie.

	Ambient	Altered	Effect	Num DF	*F*value	Pr > *F*
	mean ± se	mean ± se				
Total cover	143.7 ± 2.1	151.6 ± 2.8	**Trt**	1	13.2	**0.0009**
			**Yr**	15	20.8	**<0.0001**
			Trt x Yr	15	1.7	0.06
Grass cover	105.9 ± 1.5	102.2 ± 1.2	Trt	1	5.0	0.07
			**Yr**	15	5.4	**<0.0001**
			Trt x Yr	15	1.1	0.3
Forb cover	37.8 ± 1.7	49.4 ± 2.4	**Trt**	1	30.1	**<0.0001**
			**Yr**	15	12.5	**<0.0001**
			Trt x Yr	15	0.7	0.9
Total richness	11.2 ± 0.2	12.3 ± 0.2	**Trt**	1	21.2	**<0.0001**
			**Yr**	15	1.8	**0.04**
			Trt x Yr	15	0.4	1
Grass richness	5.3 ± 0.1	5.9 ± 0.1	**Trt**	1	20.6	**<0.0001**
			**Yr**	15	2.8	**0.0007**
			Trt x Yr	15	0.4	1.0
Forb richness	5.9 ± 0.1	6.4 ± 0.2	**Trt**	1	7.0	**0.009**
			Yr	15	1.6	0.1
			Trt x Yr	15	0.3	1.0

Trt = treatment, Yr = year, se = standard error.

**Table 2:** Summary statistics describing treatment differences on community composition and stability of tallgrass prairie.

Population Stability	**Trt**	**mean**		
	ambient	0.66		
	altered	0.68		
		Num DF	*F* value	Pr>*F*
		1	0.0	0.876
Community Stability				
*forb*	Trt			
	ambient	0.39		
	altered	0.48		
		Num DF	*F* value	Pr>*F*
		1	5.2	0.149
*grass*	Trt			
	ambient	0.49		
	altered	0.53		
		Num DF	*F* value	Pr>*F*
		1	3.7	0.195
*total*	Trt			
	ambient	0.31		
	altered	0.36		
		Num DF	*F* value	Pr>*F*
		1	5.5	0.145
Temporal Rate of Change	Trt	mean slope		
	ambient	2.84		
	altered	2.96		
		Num DF	*F* value	Pr>*F*
		1	0.0	0.87
Mean Rank Shift	Trt	mean	*r*^*2*^	
	ambient	0.52	0.05	
	altered	0.73	0.13	
		Num DF	*F* value	Pr>*F*
		1	0.9	0.342

Trt = treatment.

**Table 3:** Average abundance of dominant species over all years in ambient and altered rainfall treatments, and cumulative contribution of each species to differences in community structure between rainfall treatments based on SIMPER analysis.

**Species**	**Ambient avg. abundance**	**Altered avg. abundance**	**Cumulative contribution**
1997–2012			
*Andropogon gerardii* (grass)	72.3	66.8	0.21
*Sorghastrum nutans* (grass)	21.8	16.9	0.36
*Solidago canadensis* (forb)	17.2	15.0	0.49
*Solidago missouriensi* (forb)	1.0	12.2	0.59
*Andropogon scoparius* (grass)	1.8	6.9	0.64
2007			
*Andropogon gerardii* (grass)	77.9	70.4	0.16
*Solidago canadensis* (forb)	21.0	20.7	0.31
*Sorghastrum nutans* (grass)	27.6	19.5	0.41
*Solidago missouriensi* (forb)	0.8	14.1	0.52
*Andropogon scoparius* (grass)	3.1	11.7	0.60
2009			
*Sorghastrum nutans* (grass)	50.4	32.0	0.18
*Andropogon gerardii* (grass)	56.9	55.4	0.35
*Solidago canadensis* (forb)	32.8	25.8	0.50
*Solidago missouriensi* (forb)	2.7	22.1	0.64
*Helianthus rigida* (forb)	3.1	6.0	0.69
