# Supplementary material for: Altered rainfall patterns increase forb abundance and richness in native tallgrass prairie
Source: Sci Rep. 2016 Feb 1;6:20120. doi: 10.1038/srep20120 (PMC4735582; doi:10.1038/srep20120)
Supplement: Supplementary Information [file srep20120-s1.pdf]

Altered rainfall patterns increase forb abundance and richness in native tallgrass prairie

Sydney K. Jones[1], Scott L. Collins[1], John M. Blair[2], Melinda D. Smith[3,4], Alan K. Knapp[3,4]

Figure S1. Growing season mean soil water content (**a**; % volume water content at 0-15 cm) between ambient and altered treatments (fewer, larger rain events), and ambient total growing season rainfall (**b**; mm) between 1997 to 2012. Error bars denote one standard error. Reference lines correspond to the mean annual precipitation for all 15 years. Soil water content was significantly lower in altered treatments compared to ambient during the growing season ($P = < 0.0001$).

Figure S2. Within season soil water content (% soil water content at 0-15 cm) and precipitation by event (mm) during the 2005 growing season. Ambient treatment is shown in the top panel and altered treatment (fewer, larger rain events) in the bottom panel. Soil water content (SWC) is represented by the bold colored line, and precipitation, represented by gray bars, is shown by rain event frequency and amount. Maximum SWC was lower by 6%, and the number of days with low SWC was greater by 79% in the altered treatment compared to ambient. Over the growing season, the average deviation from ambient was 1.7%.

Figure S3. Correlations between soil water content (SWC) variability (mean/sd) on cover of dominant species over time. Dominate species were determined from the SIMPER analysis

(Table 3.). SWC variability was more predictive of plant cover than mean SWC. Cover of *Andropogon gerardii* (grass) was significantly related to year and SWC variability (P = 0.01 and P = 0.03 respectively), cover of *Sorghastrum nutans* (grass; P = 0.0001), *Solidago canadensis* (forb; P = 0.0007), *Solidago missouriensi* (forb; P = 0.0009) and *Helianthus rigida* (forb; P = 0.02) were significantly related to year but not variability in SWC. Cover of *Andropogon scoparius* (grass) was unrelated to year or SWC.

Figure S4. Correlations between soil water content (SWC) variability (mean/sd) on total cover of dominant grasses and forbs over time. Dominant species were determined from the SIMPER analysis (Table 3.). SWC variability was more predictive of plant cover than mean SWC. Forbs were significant for year only (P = 0.004). Grasses were not significant for either year or SWC variability.

Figure S1.

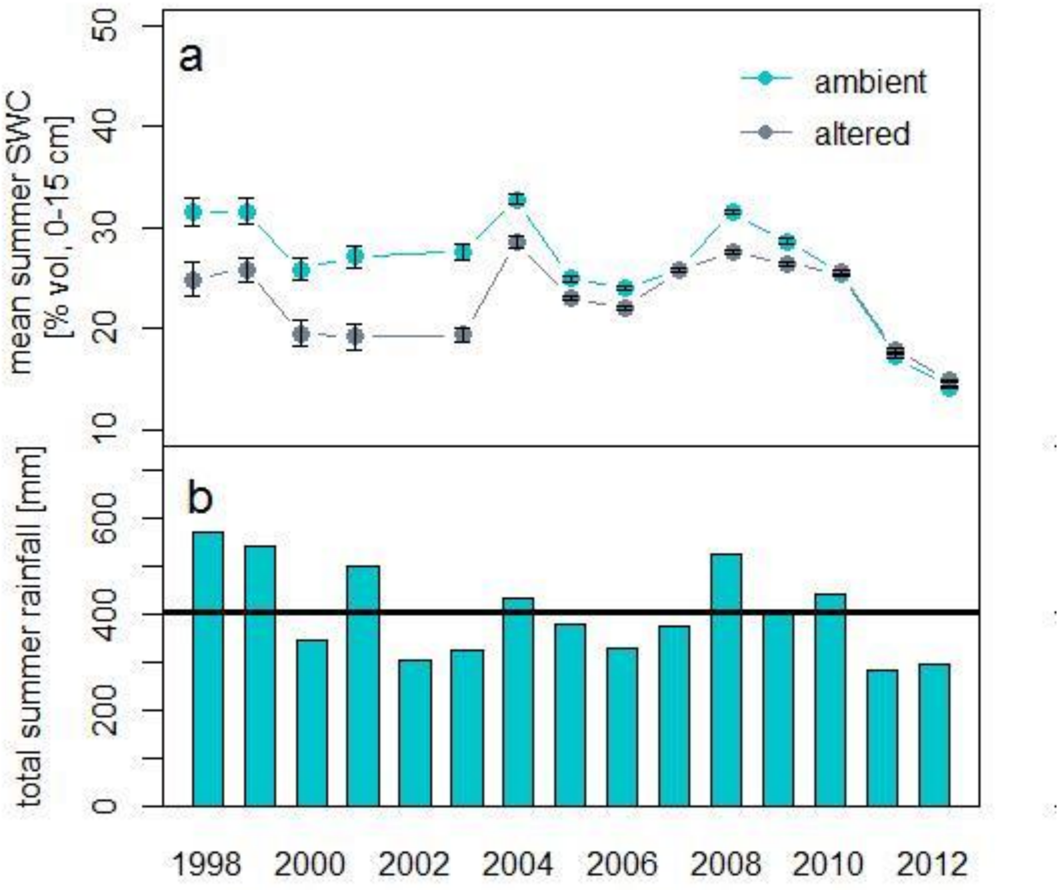

a
ambient
altered
mean summer SWC
[% vol, 0-15 cm]
10
20
30
40
50
b
total summer rainfall [mm]
0
200
400
600
1998
2000
2002
2004
2006
2008
2010
2012

Figure S2.

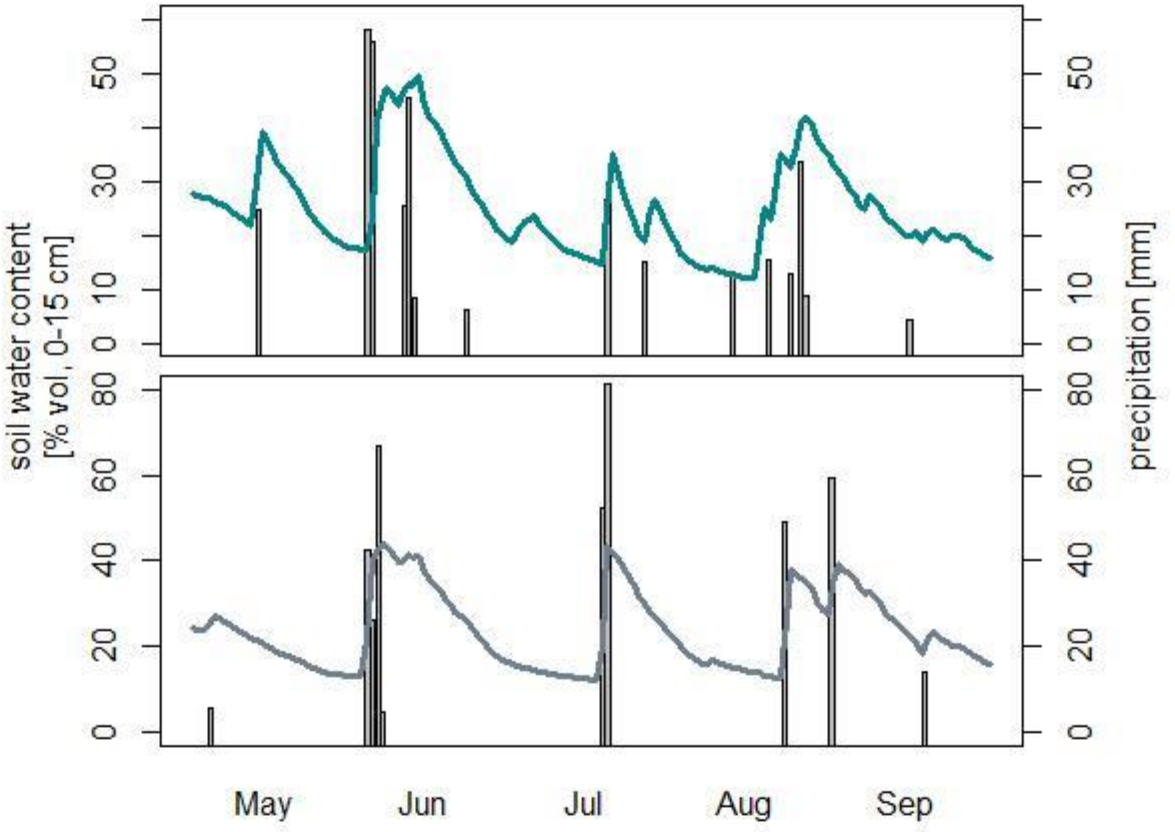

soil water content
[% vol, 0-15 cm]
precipitation [mm]
0
10
30
50
0
20
40
60
80
May
Jun
Jul
Aug
Sep

Figure S3.

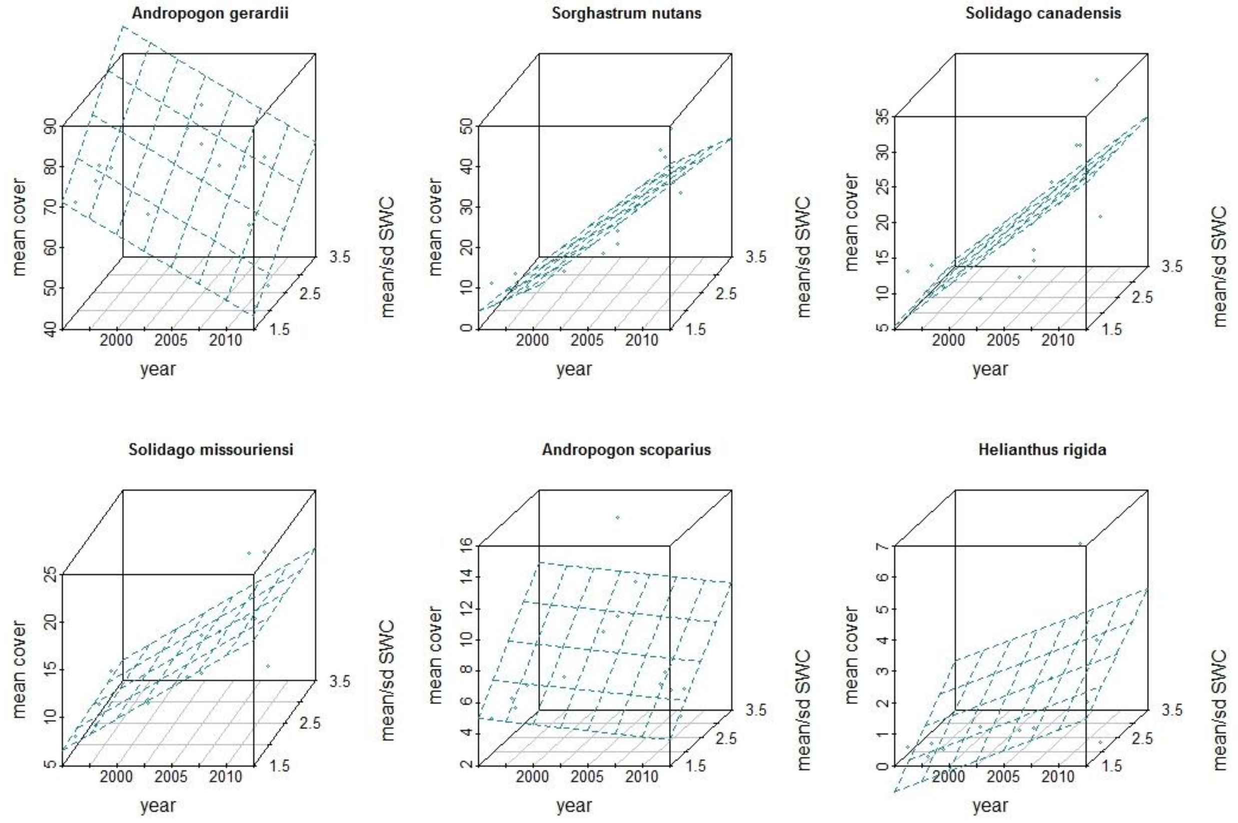
Andropogon gerardii
Sorghastrum nutans
Solidago canadensis
Solidago missouriensi
Andropogon scoparius
Helianthus rigida
mean cover
year
mean/sd SWC
2000
2005
2010
1.5
2.5
3.5

Figure S4.

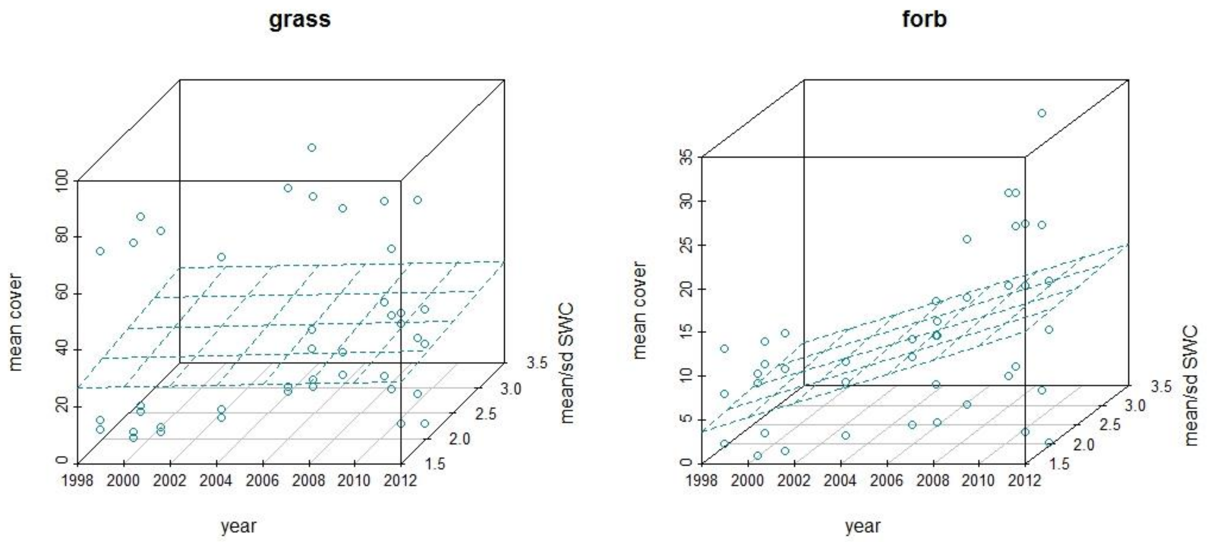

Table S1. Correlation statistics of forb richness against precipitation and SWC parameters.

	**total seasonal rainfall**	
	r^2	*P*
ambient	0.05	0.4405
altered	0.02	0.6186
	**avg. days between events**	
	r^2	*P*
ambient	0.10	0.2532
altered	0.10	0.2591
	**avg. seasonal SWC**	
	r^2	*P*
ambient	0.42	0.0127
altered	0.20	0.1054
	**SWC CV**	
	r^2	*P*
ambient	0.21	0.1027
altered	0.22	0.0904
